# Hospital and Institutional News

**Published:** 1919-03-15

**Authors:** 


### HOSPITAL AND INSTITUTIONAL NEWS.

### NURSES IN DENTAL CLINICS.

Coming at a time when quack dentistry is under full observation and discussion, the suggestions put forward at a recent meeting of the School Dentists' Society claim particular attention. By some it is advocated that specially trained nurses be placed on the staff of each children's clinic under the control of the dentist. Few deny her possible usefulness, but there is considerable disagreement as to the scope of her work. It may vary between simple care of and instruction concerning the unclean mouth to the extreme of doing extractions and fillings for the children. To us it appears that if she is to do anything more than can be described as "dressing" to the operations of the dentist, she should have a very complete training indeed, and be definitely certificated as to her abilities. Nevertheless, highly-trained dental nurses could do much to relieve the spadework of routine examination and classification of cases. The capabilities of the trained hospital nurse are sufficiently well known to give an immediate idea of what may be expected from the trained dental nurse—and the work of both should have corresponding scope. If practical dental work be done, then it seems that actual training and qualification for the same are essential. There is no doubt that these women could be trained to a higher standard of usefulness than at present, and the extremists, as in most similar discussions, tend to call up much opposition which would never arise against an efficient dental nursing service of normal limitations.

### VOLUNTARY HOSPITALS AND SUBSIDIES.

Since Sir Napier Burnett, when addressing the meeting of doctors at Newcastle-on-Tyne, so forcibly raised the question of State aid for voluntary hospitals, his widely published remarks have been re-echoed at more than one annual meeting. The Chairmen of the Royal Hospital and Royal Infirmary, Sheffield, on behalf of which institutions a joint appeal for £100,000 is being issued, have expressed their views on the subject to a representative of the press. Mr. Philip K. Wade, the Chairman of the Royal Hospital, would like to see the voluntary system kept alive, with either State or municipal aid to help it out, the body giving the aid to have some control in the management. He is not in favour of nationalisation, being quite convinced that the present management can carry on the institutions better and at less cost than either State or municipal management would. Mr. H. W. Bedford, the Chairman of the Royal Infirmary, expresses a similar view, and thinks that adequate extensions must be followed by subsidies. The worst of the subsidy idea, as discovered in Australia, is that the giving public lose interest, or their interest is no longer evidenced by gifts, and the charitable income of hospitals has a tendency to sag away. Perhaps the capitation-grant system will be the solution: the Government department or municipality responsible for certain patients paying for the cost of their maintenance when in hospital. There will still be left over a large number of patients, help to whom in time of sickness will afford a useful outlet for the friendliness of the charitable public.

### A MATRON-OF-ALL-WORK.

A very interesting document has been issued by the medical staff of the Teddington and Hampton Walk Cottage Hospital, in which the staff urges the necessity for new buildings, and details with inspiring frankness the present defects. The statement, which takes the form of a report to the chairman and his committee, is really of historical interest, for it marks a stage in the development of the cottage hospitals which is already, generally speaking, a thing of the past. We reproduce the following: "The time has come when we must express our opinion that it is neither fair to us and our work, nor to those who entrust their health and their lives to our care, that we should continue . . . in a hospital which is confessedly and hopelessly out of date; which is in many respects insanitary; which is wanting in convenience and labour-saving appliances and arrangements, and in professional equipment; and in which the nursing duties of the matron are harassingly interfered with by the necessity of performing housekeeping, bookkeeping and clerical work, much of which ought to be in charge of a clerk. The hospital site has been so built around that it is no longer suitable for its function. We are of opinion that it would be better to have for a time a modern hospital with fewer beds—if such a reduction of numbers should unfortunately be necessary for financial reasons—rather than to carry on as at present." The committee is at one with the staff; a site seems to have been found, but no active steps appear to have been taken to raise the money to buy it.

### HOSPITAL EXPANSION AT EXETER.

It has been decided to build a new wing at the Royal Devon and Exeter Hospital, first for the use of discharged sailors and soldiers, and, ultimately, for the treatment of young children and to meet the growing needs of the ear, nose, and throat department. Mr. Harbottle, the architect, has presented a report on the matter. An appeal for £20,000 will be issued. Mr. Shirley Steele Perkins has suggested that since the City Council has adopted the Child-Welfare Act and is therefore bound to provide hospital treatment for children under five, this matter should be considered in connection with the new wing. The Council would naturally come to the hospital, and the two schemes seemed to him to have a natural birth between them. The idea is a good one, and we hope that it will be carried out. It is interesting to note that Exeter is another of the county towns which is about to organise workpeople's subscriptions and to add their representatives to the committee.

### A HOSPITAL SUPERINTENDENT RETIRES.

One of the most engaging and devoted workers for the voluntary hospitals, Mr. Francis H. Moore, the general superintendent and secretary of the Royal Infirmary, Liverpool, is about to relinquish the important office which he has held for thirty-three years. Previous to this he was for ten years at St. George's Hospital, London, and he must be one of the oldest, if not the oldest, hospital secretary living who was in full work up to the end of 1918. Mr. F. H. Moore is the possessor of a charming personality, and his love of music, of which he has an unusual knowledge for an amateur, has been a source of great comfort and strength to himself, we are confident. His devotion to his hospital, and the valuable services he has put into the work during the last thirty-three years, are an example which we hope may be followed in the future by many other successful workers. The amount of superannuation granted to Mr. Moore on his retirement is not stated, but we are hopeful that it may prove as handsome and adequate as his great services undoubtedly entitle him to receive. We shall miss him greatly in connection with our work for voluntary hospitals up and down the country, and can only hope that Mr. Moore's retirement will be accompanied by sound health and afford him every opportunity to pursue the promotion for many years to come of many objects in which he is interested. We should welcome further particulars of the development, growth, and extension of the Liverpool Royal Infirmary during the thirty-three years of Mr. Moore's superintendence.

### HOSPITALS AND THE LOCAL PUBLIC.

The great majority of the hospital public, and in fact a great many of all who have dealings with hospitals, know relatively little of the medical staff and senior officers within them, but they do know what kind of porters, telephone operators, and inquiry office clerks are to be found at the outer entrance and office establishments. It is not too extreme to say that the local reputation of a hospital is considerably influenced by the behaviour of these accessory employees. A recent article in the *Modern Hospital* (Chicago) very clearly expresses the appreciation of this fact in the United States, and there as much care is apparently devoted to the links between the inner hospital and the people as to the operating-theatres themselves. No trouble is spared to secure a department and workers with the maximum possible knowledge concerning all the various activities of the institution. The more they know of the hospital and its personnel the more valuable they become, and the superintendent who exercises care and judgment in the selection and training of these people is rendering a real service to his hospital.

### INDIRECT LIGHTING IN WARDS.

The Americans are always to the fore in experiment with hospital fittings and ward equipment and, although we may not agree with all their innovations, there is one system to which we might well devote attention—namely, the indirect lighting of wards. There is nothing so distressing to a really sick man as the steady metallic glare of a metal filament lamp, and the sight of the chronic or convalescent patients leaning in uncomfortable positions to obtain the light of a centrally hung globe on a paper or piece of needlework is a sight only too common in English hospitals. The portable stand lamp and wall plug is always available for use at dressings or minor operations when a strong direct light is needed, and there seems no doubt that the diffused rays of the shielded light are both ample and the most restful for general use. American journals describe numberless excellent systems having the qualities of efficiency, cheapness, cleanliness, and adaptability to varying requirements at each particular bedside. Many of our hospital constructors would do well to consider these methods.

### DEPUTATION OF PATIENTS.

Discontent over the delays consequent upon demobilisation has affected not only military camps but military hospitals. According to the *Manchester Evening News*, a deputation of patients from the Hope Auxiliary Military Hospital, Manchester, lately visited the editor and laid before him their complaints of delay. Their complaints were published, and the authorities of the 2nd Western General Hospital were asked if they could throw any light upon the matter. They disclaimed responsibility for the delay, on the ground that certain essential Army Forms had been unobtainable in spite of repeated applications. Matters are said to have improved since. We hope they have. But with every sympathy for the men, we doubt whether any organisation can keep pace with the natural impatience of the gigantic numbers concerned. At all events, a deputation to a newspaper is preferable to a mad riot.

### METROPOLITAN BOARD'S GOOD BARGAIN.

Trouville Military Hospital has been acquired by the Metropolitan Asylums Board from the War Office, and will be brought to England for use as a sanatorium. The hospital, which cost the War Office £150,000, has been obtained for £5,000, including equipment. The purchasing body, however, will pay the cost of the removal. Where it will be re-erected has not yet been finally established. Before the war the Metropolitan Asylums Board acquired sites for sanatoria at East Grinstead, Basingstoke, and near Godalming, and one of these will be chosen. In pre-war days the estimates for the building of the sanatoria were between £30,000 and £40,000 each. As the Trouville hospital is described as eminently suited for its new purpose, it is clear the Board has made a good bargain.

### A PIONEER OF HEALTH VISITING.

With the recent retirement of Dr. A. Bostock Hill from the post of Medical Officer of Health for Warwickshire, the pioneer of health visitors leaves active public work. After his appointment in 1876 he decided that home visiting must be undertaken and appointed a woman to this then new sphere of work. When it was urged that the Local Government Board might not sanction the appointment, Dr. Hill said that he could appoint them as clerks and then send them on rounds of visiting. As it happened the appointments were quickly recognised, and the county likes to refer to his organisation as the Warwickshire system, which was soon followed in other counties.

### NATIONAL RELIEF FUND'S RESERVE.

THE National Relief Fund had, to the end of September 1918, collected £6,437,733, and disbursed £3,088,929, for naval and military relief, and £808,952, for civil relief. There was in hand, therefore, a sum of over two-and-a-half millions, and as, apparently, there are very few current out-goings, except a moderate amount of relief to the East Coast towns, the funds are probably increasing every day. It would be interesting to know what is in the mind of the committee of the fund as to the ultimate use of this substantial sum. Seemingly, the State has taken over much relief work through the out-of-work donation system, and by cheapening the price of bread and increasing the old age pensions. We do not advocate spending for spending's sake, but we are conscious of the presence within the community of persons and institutions who have been very badly hit indeed during the war, but have not come within the ken of the National Relief Fund. It is possible that the Fund is in need of a good information department.

### STERILISATION OF ANTHRAX WOOL.

LECTURING at Bradford last week, Dr. T. M. Legge referred to the previous attempts at limiting the entry of anthrax into this country. The old principles were directed towards the removal of all possible diseased fleeces and blood-clots, while dust was taken out by forced ventilation. Workers handling the material received special instruction and protection against infection, a complete campaign being organised along the lines of education, notification, and immediate treatment. Nevertheless, during the war there had been a marked increase in the number of reported cases, and it was satisfactory that researches recently completed had shown the possibility of attacking the danger at its source, namely, by sterilisation of the wool itself. The most important measure was to protect the animals themselves from infection, and this was efficiently done in Australia and New Zealand, but in Northern and Central Asia the local tribes and producers could not be expected to co-operate successfully. Steam sterilisation destroyed the bacteria, but at the same time ruined the wool for manufacturing purposes, and therefore the discovery of an efficient process was more than welcome. Blood clots could first be washed out by agitation in warm water containing soap and sodium bicarbonate, followed by squeezing in heavy rollers. The partly cleansed wool was then treated with warm 2.5 per cent. formaldehyde in water, again pressed, and finally dried in a stream of heated air. Almost complete sterilisation was effected in this way, and a trial disinfecting station was now being organised in this country. Later, arrangements were to be made to establish corresponding plants in the Asiatic ports of origin and so attack the danger at its very source.

### THE CHILDREN OF SOLDIERS.

WE must heartily agree with the remarks of a correspondent in a recent issue of the *Times*, when he states that one of the most efficient remedies of the enormous wastage of young lives during the years of war is to encourage a greatly increased care for the children of our soldiers and sailors. They are the children of fighting men, men of the finest stamina and often the finest minds. They represent, on grounds of heredity, the hope of the nation. Every encouragement should be given to those returning from the war to marry at once. The pre-war economic prejudice against men with families should be forgotten, and every attempt made to aid them. Nowadays we cannot overestimate the value of the soldiers' children.

### A NEW NAME FOR AN OLD HOSPITAL.

THE South Eastern Hospital for Children is not a new hospital, but only an old one under a new name. It was founded in 1872 by Miss Edith Elwes and a few friends as a Convalescent Home for Children with accommodation for six patients. It was moved in 1886 to its present site of 1½ acres. The freehold was given by Mr. C. Gordon Hennell. It then changed its name to the Hospital and Home for Sick Children. During the last ten years it has gradually grown into a Children's Hospital, with sixty beds, which are nearly always occupied. At the recent Annual Meeting it was decided to change the name to the "South Eastern Hospital for Children," and the Committee hopes it will now be recognised as one of the children's hospitals of London. As the hospital is doing good work in a very poor neighbourhood, receiving last year 519 in-patients, and attending 7,106 out-patients, the Committee trusts that friends will come forward to clear the debt of £800 owing to the bankers.

### MEDICAL OFFICERS IN THE PULPIT.

HOSPITAL chairmen have been known to read the lessons in hospital chapels, or in churches on hospital Sunday, and even to deliver addresses to nurses from the lectern; but it is seldom that an incumbent invites a medical man into his own pulpit. It was, however, in his official capacity as medical officer of health for Huddersfield that Dr. S. G. Moore was asked by Canon Tupper Carey to occupy the pulpit of the Parish Church on Infirmary Sunday, recently. The vicar first remarked that health was a subject best left to experts. Dr. Moore then followed him and said that Huddersfield was deficient in civic pride, the pride which animated centres of learning or cities which cherished the charters that had been granted to them in the past. He then praised the welfare work of the Huddersfield Public Health Union and the achievements of the Royal Infirmary, and concluded by emphasising the part which was played by health in the happiness and well-being of mankind.

### A DOCTOR ON HOSPITAL ADVERTISING.

A thin attendance at a recent meeting of the Bradford Eye and Ear Hospital led a woman present to complain that lack of advertisement was the cause. Dr. Bronner, in support of this view, paid a compliment to the Leeds General Infirmary, which he thought owed much of its success to the prominence with which its needs were made known and the subscriptions and legacies received were advertised. Dr. Bronner was thereupon invited to make public some of the successes which the Eye and Ear Hospital had gained in the sphere of treatment. This raises the point whether a hospital is better advertised by descriptive articles, or by statements of facts and figures. On this point Dr. Bronner expressed no opinion.

### SURGICAL TUBERCULOSIS IN THE WEST.

Lately a conference has been held at Taunton to consider the establishment of an institution for the treatment of surgical tuberculosis in the West of England. The report of the proceedings reads most encouragingly to those interested in tuberculosis, for the usual faults of mutual jealousy, of want of co-operation, of isolated projects foredoomed to failure, which dog the British anti-tuberculosis campaign, were entirely absent. The conference comprised delegates from the following bodies: Somerset County Council, Devon County Council, Exeter City Council, Plymouth County Borough Council, and the Devon Insurance Committee. It was resolved to invite in addition the County Councils of Cornwall, Dorset, Gloucester, and Wilts, with the County Boroughs of Bath, Bristol, and Gloucester, to combine to provide an institution of the nature indicated, containing not less than a hundred beds. At the same time it was agreed that although a large measure of co-operation would be an advantage, none the less the institution would still be possible were two or three authorities to abstain. Of course beds in excess of the hundred could be provided in future. Dr. Savage, M.O.H., Somerset County, rightly drew attention to the better results in surgical tuberculosis obtainable at such an institution over those common in general hospitals. Indeed the inferiority of the conservative to the operative treatment of this form of the disease is now acknowledged. We hope to hear that this excellent scheme has duly progressed and prospered.

### THE DESTRUCTION OF TEHIDY SANATORIUM.

The Cornish County Tuberculosis Committee is indeed to be condoled with on the loss of their Sanatorium, which was rapidly approaching completion. With the disease as widespread as it is, however, no time need be wasted in regrets. Moreover, out of evil good may come, for in the old scheme there were many points open to criticism. A nursing staff of thirteen, for instance, for about forty-five male adult patients, was far too big. Converted country houses make uneconomical sanatoria, and we have no doubt that second thoughts will in due time produce much better results. At the meeting of the Cornwall Insurance Committee, after reference had been made to the new and unfortunate situation, one member expressed doubts of the value of sanatorium treatment, quoting the increase in the disease in the last four years. But that increase is almost worldwide and due to hardships imposed by the war. Certainly the pronounced contagionist school laid, as we can now all see, far to much stress on infection, and underrated the effect of general health and constitutional resistance as the premier anti-tuberculosis agency. But that is no reason for condemning the sanatorium, which acts by improving and restoring physiological fitness.

### CORONER STICKS UP FOR DOCTORS.

When one considers that hundreds of thousands of casualties pass through the hospitals in the course of a year it is a matter for wonder that so seldom anything in the shape of a bungle, resulting in injury to a patient, occurs. But the facts brought to light at an inquest in Bristol are clearly indicative that somebody was at fault in the case under enquiry. A child was taken to the Bristol General Hospital at about 12.15 p.m., badly scalded, and was not seen by a doctor until 1.40 p.m. The person who came with the child said that the nurses did not like to disturb the doctor, who was at lunch. The child did very well at first under treatment, but subsequently died. The Coroner, in summing up, said the hospital, like every other well-managed institution, would no doubt inquire into the matter of the seeming fault in the system. That was not a task for the jury. He, however, would stake his existence that there was not one doctor on the staff who would not have come at once, lunch or no lunch; he knew something of t[illegible] work of doctors, after 27 years' experience, and was absolutely certain as to what would be the action of a doctor in such a case.

### THIS WEEK'S DRUG MARKET.

The market continues to be characterised by the same features that have been noticeable during the past two or three months. That is to say, the downward tendency in a considerable range of drugs continues, and buyers are still disinclined to commit themselves to the placing of large orders. Nevertheless, there is more buying, for the reason that many distributors have come to the very end of their small stocks of various commodities and are therefore obliged to come on to the market in order to keep their businesses going. This accumulation of small orders gives more life to the market, which is a relief from the depressing conditions that have ruled for so long. Among the articles which are again quoted at lower rates may be mentioned benzoic acid, sodium benzoate, salicylic acid, sodium salicylate, aspirin, chloral hydrate, creosote, formaldehyde, paraldehyde, citronella oil, clove oil, phenolphthalein, potassium permanganate, and cubeb oil. It is not improbable that before long the position as regards opium will be less stringent, and rather lower prices for sandalwood oil are forecasted. The prices of menthol, peppermint oil, and camphor are firmly maintained.

